# Cochlear motion across the reticular lamina implies that it is not a stiff plate

**DOI:** 10.1038/s41598-022-23525-x

**Published:** 2022-11-04

**Authors:** Nam Hyun Cho, Sunil Puria

**Affiliations:** 1 Department of Otolaryngology-Head and Neck Surgery, Harvard Medical School, Boston, MA 02114 USA; 2 Eaton-Peabody Laboratories, Massachusetts Eye and Ear, Boston, MA 02114 USA; 3 Speech and Hearing Bioscience and Technology Program, Harvard University, Cambridge, MA 02138 USA

**Keywords:** Neuroscience, Auditory system, Cochlea

## Abstract

Within the cochlea, the basilar membrane (BM) is coupled to the reticular lamina (RL) through three rows of piezo-like outer hair cells (OHCs) and supporting cells that endow mammals with sensitive hearing. Anatomical differences across OHC rows suggest differences in their motion. Using optical coherence tomography, we measured in vivo and postmortem displacements through the gerbil round-window membrane from approximately the 40–47 kHz best-frequency (BF) regions. Our high spatial resolution allowed measurements across the RL surface at the tops of the three rows of individual OHCs and their bottoms, and across the BM. RL motion varied radially; the third-row gain was more than 3 times greater than that of the first row near BF, whereas the OHC-bottom motions remained similar. This implies that the RL mosaic, comprised of OHC and phalangeal-process tops joined together by adhesion molecules, is much more flexible than the Deiters’ cells connected to the OHCs at their bottom surfaces. Postmortem, the measured points moved together approximately in phase. These imply that in vivo, the RL does not move as a stiff plate hinging around the pillar-cell heads near the first row as has been assumed, but that its mosaic-like structure may instead bend and/or stretch.

## Introduction

The great sensitivity and frequency selectivity of mammalian hearing originates in the mechanical properties of the cochlea. Cochlear mechanical motions in response to sound are amplified using metabolic energy. The motor element of this cochlear amplification is the outer hair cell (OHC), whose length can vary at sound frequencies^1,2^. Each tilted OHC, with its attached supporting Deiters’ cell (DC) and the DC phalangeal process (PhP) extending apically from the DC, form a Y-shaped building block of the complex mechanical skeleton^3^ occupying the space between the reticular lamina (RL) and basilar membrane (BM) within the organ of Corti (OoC). How the OHCs, acting through this skeleton of Y-shaped structures, work to achieve cochlear amplification is not fully understood.

In the classic view of the cochlea, BM motion was considered to be the most important factor, and the motions of the rest of the OoC were thought to follow the BM motion^4,5^. However, recently developed optical methods have allowed motion measurements within the OoC of live, sensitive animals, and these measurements have revealed a much different picture. In response to sound, the motions of other structures within the OoC are much larger and are amplified over a larger frequency range than those of the BM^6–11^.

Another recent development is the realization that the phasing of the OHC motion required to amplify BM motion does not arise from a resonance in the tectorial membrane (TM)^9,12,13^, but instead arises from an unexpected phase of the radial motion of the RL. The RL radial-motion phase measured by Lee et al.^9^ was almost opposite to the phase expected from the OoC rotation resulting from BM motion. How such an RL phase might be produced is unknown. The RL surface can be likened to a *mosaic*, in that it is comprised of a repeating pattern of OHC cuticular plates and DC PhPs, all held together by adhesion molecules^14,15^, and as such it may bend and/or stretch. It seems possible that the skeleton of Y-shaped structures below the RL mosaic may play a role in creating the required RL radial-motion phase. The longitudinal and radial angles of the PhP branches of the Y-shaped structures vary across the three rows of OHCs^3^ and this might cause the motions to be different across the three OHC rows, but this has yet to be experimentally or computationally demonstrated.

Most past work that measured motion in the OoC has concentrated on the motion profile along the direction corresponding to the OHC length change from electromotility, which is the direction in which the OHC force acting against the BM is expected to produce BM cochlear amplification^16^. Here we examine OoC motion at various depths along this direction, and also at different radial locations corresponding to each of the three OHC rows. The results add to our understanding of how the OoC works, and may shed light on the mechanisms by which the RL radial motion can achieve the correct phase required for cochlear amplification.

## Results

**Figure 1:**
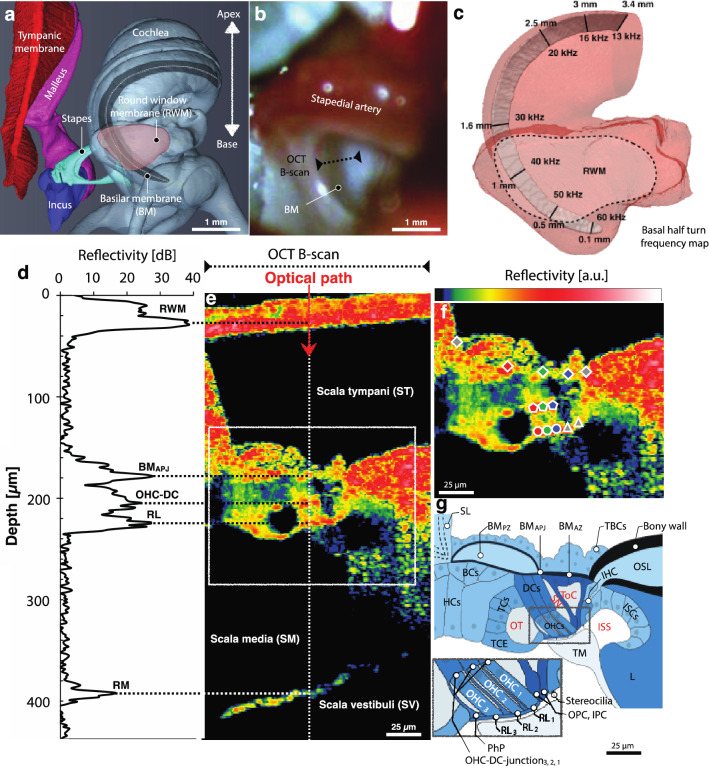
The anatomy of the gerbil ear and in vivo cochlear imaging. (**a**) A micro-computed-tomography (µCT)–based 3D reconstruction shows the main structures of the gerbil middle ear (i.e., tympanic membrane, malleus, incus, and stapes), and the cochlea (base to apex) with the round-window membrane (RWM; pink region) and internal basilar membrane (BM; dark-gray region) indicated. (**b**) A photograph (specimen G504) shows the RWM region through which 2D “B-scan” cross sections of the BM in the basal “hook region” of the cochlea can be imaged using optical coherence tomography (OCT; black dotted line). (**c**) The frequency map of the BM “basal half turn” (from panel **a**) is shown as a function of distance from the basal tip. (**d**) An example of the depth profile (backscattered-light reflectivity) of a single 1D “A-scan” (white dotted line in (**e**) shows several peaks corresponding to: the RWM; the junction (BM_APJ_) of the BM arcuate zone (BM_AZ_) and pectinate zone (BM_PZ_); the junction (OHC-DC) of an outer hair cell (OHC) and Deiters’ cell (DC); the reticular lamina (RL); and Reissner’s membrane (RM). (**e**) An in vivo OCT B-scan image (G612), as measured through the RWM. (**f**) Enlarged view of **e** detailing the organ of Corti (OoC) structure and marking the following OCT-vibrometry measurement points (left to right and top to bottom): outer BM edge, BM_PZ_, BM_APJ_, BM_AZ_, inner BM edge (gray/red/green/blue/gray diamonds); OHC-DC-junction_3,2,1_ (red/green/blue pentagons); RL_3,2,1_ (red/green/blue circles); and outer and inner pillar cells (OPC and IPC; gray triangles). (**c**, **g**) Drawing by Andrew A. Tubelli. (**g**) A labeled cross-sectional drawing of a representative OoC structure. The inset details the three rows of OHCs where they meet the RL at their apical ends, as well as the OPC, IPC, and inner-hair-cell (IHC) stereocilia. Other abbreviations: SL, spiral ligament; TBCs, tympanic border cells; BCs, Boettcher cells; OSL, osseous spiral lamina; HCs, Hensen’s cells; TCs, tectal cells; TCE, tectal-cell extension; ISCs, inner-sulcus cells; TM, tectorial membrane; L, limbus; OT, outer tunnel; SN, Space of Nuel; ToC, tunnel of Corti; ISS: inner spiral sulcus; PhP, phalangeal process.

The location where vibration measurements were made, relative to the anatomy in the hook region of the gerbil cochlea, is shown in Fig. 1a–c. At the wavelength of our optical coherence tomography (OCT) system, reflectivity peaks suitable for making vibrometry measurements were observed at the RL, at the bottom of each OHC at its junction with the DC (OHC-DC-junction), and at the BM (Fig. 1d–g). At the RL and OHC-DC-junction points, it was often possible to see separate areas of high reflectivity that seemed to be aligned with each of the three OHC rows. The second-row area of high reflectivity was sometimes best seen at a slightly different longitudinal location, thus requiring a longitudinal offset (7–15 μm) for row-2 measurements relative to those of rows 1 and 3.

### Motion measurements and gains

**Figure 2:**
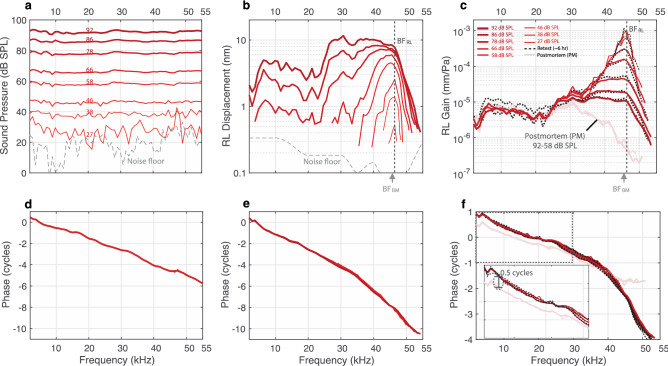
In vivo RL_3_ vibrometry measurements at different stimulus levels in the hook region of the gerbil cochlea (G637). (**a**) The ear-canal sound pressure levels of pure tones used to stimulate the specimen, ranging from an average of 27 to 92 dB SPL (line thickness and darkness increase with level), as well as a noise-floor reading (gray dashed line). (**b**) RL_3_ displacements as functions of frequency, corresponding to the different stimulus levels in panel (**a**). Displacement measurements were rejected at frequencies where their magnitude was less than 6 dB above the noise floor (gray dashed line). The best frequency (BF) for the RL_3_ location (BF_RL_) was 46.4 kHz (gray dotted vertical line), and for the BM_APJ_ location (BF_BM_) was 45.6 kHz (gray arrow below the panel). (**c**) The in vivo (dark red) and postmortem (PM; faded red) RL_3_ displacements were normalized by the stimulus sound pressure to produce the RL Gain (in units of mm/Pa). The black dotted lines indicate repeated in vivo measurements made ~ 6 h later. (**d**–**f**) The respective phase responses of the sound pressure, RL_3_ displacement, and RL_3_ gain. (**f**) An enlarged view of the RL_3_-gain phase from the black-dotted region of the panel is shown.

An example of motion displacement measurements from RL_3_ (the RL point at the top of OHC_3_, in the third OHC row), along with the corresponding ear-canal sound-pressure measurements, are shown in Fig. 2. Also shown is the displacement/sound-pressure ratio, which we refer to as the motion “gain.” In all later figures, the motion displacement measurements are expressed in terms of their gain, which includes both a magnitude and a phase.

**Figure 3:**
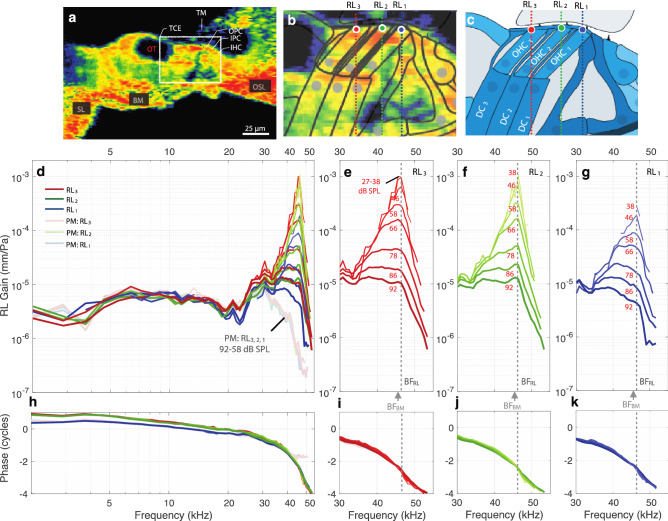
The in vivo and PM RL Gains across the apical ends of the three rows of OHCs (RL_3_, RL_2_, RL_1_) from gerbil G637. (**a**) A 2D cross-sectional OCT image of a representative in vivo gerbil OoC with labeled key structures. Note that the OoC structure is displayed upside down in comparison to Fig. 1. (**b**) An enlargement of the region in panel **a** within the white box, with overlaid line drawings of the cells and other structures, and details of the measurement locations across the apical ends of the three rows of OHCs (RL_3_, red circle; RL_2_, green circle; and RL_1_, blue circle). Each colored vertical line indicates the lateral position and direction of the different OCT A-scans for the OCT vibrometry measurements. (**c**) A labeled cross-sectional drawing from the 2D OCT image in panel (**b**). (**d**) In vivo (dark red/green/blue) and PM (faded red/green/blue) gains for RL_3_, RL_2_, and RL_1_, respectively. (**e**–**g**) The in vivo, active-amplification region showing RL_3_, RL_2_, and RL_1_ gains, respectively. The gains were highest at low SPLs. The numbers in panels (**e**–**g**) indicate the stimulus level for each gain response (in panel **e** the curves overlap for 27–38 dB SPL**)**. (**h**–**k**) The phase responses corresponding to panels (**d**–**g**). In this figure the displacements were normalized by the sound pressure to produce gains in units of mm/Pa, and the phase responses are in units of cycles. The frequency axis is on a log scale in panels (**d**) and (**h**), but on a linear scale in panels (**e**–**g**) and (**i–k**). (**b**) Overlaid line drawings and (**c**) cartoon drawing by Andrew A. Tubelli.

**Figure 4:**
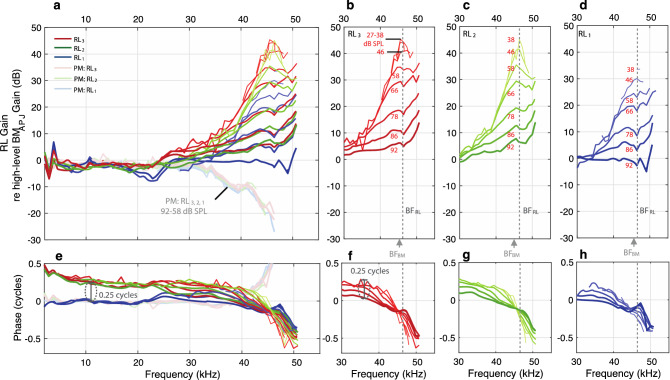
The in vivo and PM RL gains, normalized by the BM_APJ_ gain (measured at a high stimulus level), in order to remove the effects of the cochlear traveling wave, for specimen G637. (**a**) Direct comparisons of the in vivo and PM RL_3, 2, 1_ normalized gains (in dB). (**b–d**) In vivo normalized gains in the near-BF region for RL_3_, RL_2_, and RL_1_, respectively. (**e–h**) The phase responses corresponding to panels (**a–d**), respectively. Note that the baseline high-level BM_APJ_ gain used for normalization was calculated as the average of ten measurements made at 92 dB SPL. All frequency axes in this figure are on a linear scale.

Figure 2 shows that as the sound level increased, the RL_3_ motion grew compressively at frequencies near the best frequency (BF), but at low frequencies (< 20 kHz) it grew close to linearly (i.e., the gains stayed the same, e.g., Figs. 2c, 3d, 4a). At BF, the RL motion had more than 60 dB of gain relative to the motion in the dead animal (Fig. 2c). This extraordinarily high gain demonstrates that this was a sensitive preparation. Repeated measurements made at the same location indicate that the in vivo specimen was stable even after 6 h (Fig. 2c, dotted black and solid red lines). There was little variation in phase across sound levels (the red lines overlap), but the phase did change from living to dead (Fig. 2f and enlarged inset Figure). At low frequencies there was almost a reversal of the RL_3_-motion phase from living to dead.

### Motions across the tops of the three OHC rows (RL_1–3_)

The motions in one animal at the points on the RL surface corresponding to the apical surface of each of the three rows of OHCs (RL_1_, RL_2_, and RL_3_) are shown in Figs. 3, 4. The gains in the living and dead animal, along with OCT images of the anatomy from the same living animal, are shown in Fig. 3. To help remove the contribution to RL motion of the BM traveling wave, we normalized the RL gain by the BM gain (Fig. 4) at the junction between the arcuate and pectinate zones (BM_APJ_). For this, the BM gain was obtained by averaging multiple measurements made at a high stimulus level where there was relatively little cochlear amplification. RL results from additional animals are shown in Supplementary Figs. 2–4.

The data from all three RL rows show similar patterns of compressive nonlinear growth near BF, linear growth at low frequencies, and phases consistent with drive from a traveling wave (Fig. 3). The BF for the RL (BF_RL_) was typically higher than that for the BM (BF_BM_). In this animal, at frequencies near BF_RL_, the motion amplitudes at RL_2_ and RL_3_ were similar, but the amplitude at RL_1_ was ~ 15 dB less (Figs. 3e–g, 4b–d). In contrast, below 20 kHz there was very little difference in the magnitudes of the RL gains, and furthermore, the magnitudes were similar in the living and dead animal (Figs. 3d, 4a). However, in the phase at low frequencies, RL_1_ had the same phase for the living and dead animal, whereas RL_2_ and RL_3_ had a phase advance of more than 0.25 cycles in the living animal compared to the dead animal or to RL_1_ (Fig. 4e).

Removing the traveling-wave phase (by normalizing by the high-level BM_APJ_ gain) and plotting frequency on a linear scale allow the phase differences (and thus group delays) of RL_1–3_ to be seen more easily (Fig. 4). Near BF_RL_ and down to 30 kHz, all three RL rows have phase patterns in which lower levels show a steeper and greater phase *advance* than higher levels (Fig. 4f–h; lower-level lines are thinner and have lighter colors). Above BF_RL_, the continuation of this pattern results in a greater phase *delay* at lower levels. For all three RL rows, the group delays (the negative slopes of the phase-versus-frequency functions in Fig. 4f–h) became smaller as the sound level increased (Fig. 4f–h), which is consistent with the RL tuning becoming wider at higher sound levels. Interestingly, at each RL row the phase-versus-frequency curves from different sound levels cross at a frequency slightly lower than BF_RL_ (like the pattern reported for BM motion^4^). The phase-crossing frequency was similar for RL_2_ and RL_3_, but was slightly lower for RL_1_ (Fig. 4f–h). Another interesting feature of the data in Fig. 4 is that at high levels the RL gain relative to the high-level BM_APJ_ gain was largest at a frequency that was above the BFs of the BM and RL. We will return to this in the Discussion.

**Figure 5:**
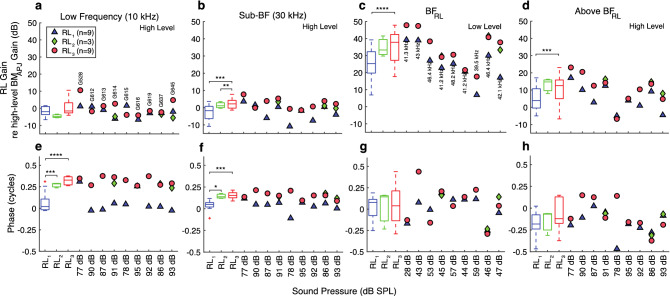
Summary comparisons across animals of the in vivo normalized gains at the apical ends of the OHC rows (RL_3, 2, 1_), for selected frequencies. (**a–d**) The respective normalized-gain magnitudes at a low frequency (10 kHz, high stimulus level), near 30 kHz (Sub-BF, high stimulus level), at BF_RL_ (low stimulus level), and above BF (high stimulus level). The above-BF_RL_ frequencies used in panels (**d**) and (**h**) correspond to the high-stimulus-level magnitude peak for each animal. (**e–h**) The phase summaries corresponding to panels (**a–d**), respectively. The individual datapoints in columns 1–2 were averaged over a 1/3-octave width centered around 10 and 30 kHz, respectively, while those in column 4 were averaged over a 1/6-octave width centered around the selected frequency. The points shown in column 3 each represent the value at a single frequency. Note that the stimulus levels listed on the horizontal axes and BF values labeled in panel **c** vary across animals (labeled in panel **a**). The box-and-whisker plots on the left-hand side of each panel provide statistical summaries of the individual results, matched by color. The ANOVA *p*-values between categories are indicated by horizontal brackets at the top. The number of asterisks above each bracket corresponds to the following degrees of statistical significance: *p* < 0.05 (*), *p* < 0.02 (**), *p* < 0.006 (***), and *p* < 0.001 (****), all of which are of greater significance than the *p*_0_ = 0.05 criterion. Insignificant *p*-values are not displayed. The red ‘ + ’ symbols indicate outliers, the boxes indicate the interquartile range (IQR) with 95% confidence, and the horizontal line inside each box indicates the median value. The whiskers indicate the minimum and maximum of the range (excluding outliers). In some cases the RL_2_ whiskers are too small to be visible. The RL_3, 2, 1_ results for animal G637 are shown in Figs. 3 and 4.

The RL gains relative to the high-level BM_APJ_ gains, compared across animals, are shown in Fig. 5. Across horizontal-axis positions in Fig. 5, summary statistics are shown as box plots and data points from each animal are shown as individual symbols, with the same animal order in all panels (and in later summary figures). From left to right, each panel summarizes data from a different frequency, with the magnitudes in the top row and phases in the bottom row. The results in Fig. 5 correspond to the highest available stimulus level common to all of the structures in a given animal, except for the third column, in which the results correspond to the lowest available level. At a representative low frequency (10 kHz), all but one animal had RL gains within 10 dB of the high-level BM_APJ_ gain, and most RL_3_ gains were higher than the RL_1_ gains (Fig. 5a). All of the RL_2_ and RL_3_ phases were advanced relative to the BM_APJ_ phase by close to 0.25 cycles, but 7 of 9 RL_1_ phases were close to the BM_APJ_ phase (Fig. 5e). At 30 kHz (Fig. 5b, f), which is close to 0.5 octaves below BF_RL_, most RL gains were within 5 dB of the high-level BM_APJ_ gain, and the phases were in a narrow range between 0 and 0.25 cycles of the BM_APJ_ phase, with all of the RL_1_ phases less than the RL_3_ phases (Fig. 5b, f). At BF_RL_ (Fig. 5c, g) there were a wide range of gains across animals, but in all cases the gain of RL_3_ was significantly greater than that of RL_1_, on average by 10 ± 1 dB (p < 0.001, Fig. 5c). At BF_RL_, the RL_1–3_ phases were mostly within 0.25 cycles of the BM_APJ_ phase, and in each animal (except one), the RL_1_ and RL_3_ phases were close. At the above-BF_RL_ frequency, measurements of RL motion relative to BM_APJ_ motion (which were usually done at the highest frequency for which reliable measurements were available in the high-level BM_APJ_ gain used for normalization), the RL gains were consistently greater than the BM_APJ_ gain and in almost all cases had a phase lag compared to the BM_APJ_ phase (Fig. 5d, h).

### Motions of the three OHC rows at their junctions with the Deiters’ cells (OHC-DC-junction_1–3_)

**Figure 6:**
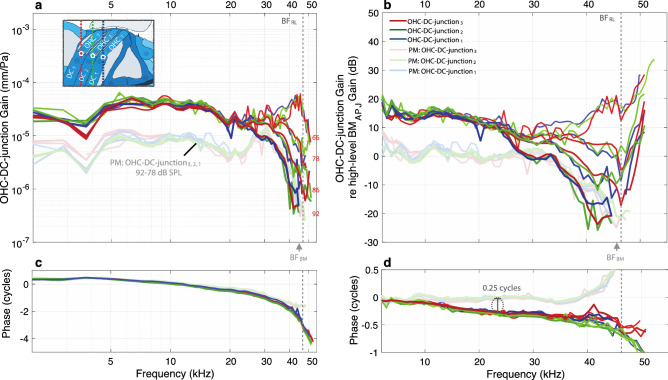
Motion comparisons of the OHC-DC junctions at the bottom ends of the OHCs, across the three OHC rows (OHC-DC-junction_3, 2, 1_), for specimen G637. (**a**) In vivo (darker colors) and PM (faded colors) OHC-DC-junction_3, 2, 1_ gains relative to the sound pressure (in units of mm/Pa). (**b**) The gains in (**a**) normalized by the high-level (92 dB SPL) BM_APJ_ gain (in dB). The inset drawing in panel (**a**) shows the OCT-vibrometry measurement locations for OHC-DC-junction_3_ (red pentagon), OHC-DC-junction_2_ (green pentagon), and OHC-DC-junction_1_ (blue pentagon). Each colored vertical line indicates the lateral position and direction of the OCT A-scans. (**c**, **d**) The phase responses corresponding to panels (**a**, **b**). The frequency axes are on a log scale in the first column and on a linear scale in the second column.

The motions in one animal across the bottoms of the three rows of OHCs at their junctions with the Deiters’ cells (OHC-DC-junction_1–3_) are shown in Fig. 6. The OHC-DC-junction gains (the displacement divided by the sound pressure) are shown on the left with a log-scaled frequency axis, and the OHC-DC-junction gains with the traveling-wave drive removed (through normalization by the high-level BM_APJ_ gain) are shown on the right with a linear frequency axis.

In the example animal, there was little difference among the gains of the three OHC-DC-junction rows at any given frequency or level (Fig. 6a, b). At frequencies near BF_RL_, all three OHC-DC-junction rows had compressive growth and were similar in gain (Fig. 6). Near BF_RL_, the three OHC-DC-junction rows had broad peaks at low sound levels, but at higher levels the amplitude decreased as the frequency increased, and above BF_RL_ it tended to decrease (Fig. 6a). At frequencies near BF_RL_ and at low sound levels, the OHC-DC-junction gain was greater than the high-level BM_APJ_ gain, but at high sound levels the OHC-DC-junction gain was less than the high-level BM_APJ_ gain (values below 0 dB in Fig. 6b). At frequencies below BF_RL_ (near 30 kHz), the gain of the OHC-DC-junction motion at the highest stimulus level was actually less than in the dead animal (Fig. 6b), which implies that the active motion was partially cancelling the passive motion. A similar pattern was seen in other animals (Supplementary Figs. 6b–8b).

At low frequencies (an octave or more below BF_RL_), the three OHC-DC-junction rows had linear growth at the levels tested, and considerable gain (~ 15 dB) relative to the OHC-DC-junction gain in the dead animal (the dark-colored lines are above the faded lines in Fig. 6a) and relative to the high-level BM_APJ_ gain (lines above 0 dB in Fig. 6b). Above BF_RL_, as the frequency increased, the OHC-DC-junction gain decreased relatively little (Fig. 6a), but the BM_APJ_ gain decreased faster so that above BF_RL_ the OHC-DC-junction gain was greater than the high-level BM_APJ_ gain (above BF_RL_ the lines go up in Fig. 6b).

The phases of the OHC-DC-junction gains were similar across the three rows at mid frequencies, but the phases deviated from each other at high and low frequencies (Fig. 6c, b). At mid frequencies the OHC-DC-junction phases lagged behind the high-level BM_APJ_ phase and the OHC-DC-junction phase of the dead animal by ~ 0.25 cycles (Fig. 6d). At the lowest frequencies, the lags in the OHC-DC-junction phase approached zero (Fig. 6d). Near the BM_APJ_ BF (BF_BM_), the OHC-DC-junction_3_ phase lagged behind the high-level BM_APJ_ phase by ~ 0.25 cycles, with rows 1 and 2 lagging more.

**Figure 7:**
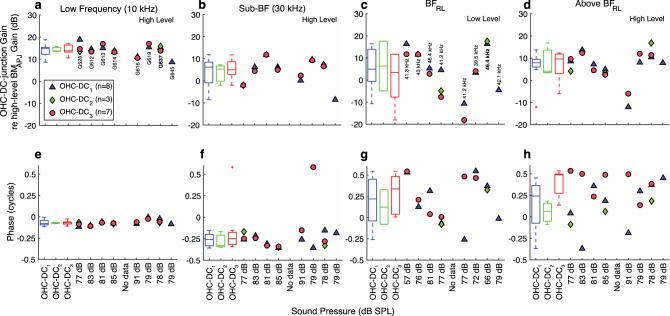
Summary comparisons of the in vivo normalized OHC-DC-junction gains at the basal ends of the OHCs, across animals, for the three OHC rows (OHC-DC_3,2,1_). (**a**–**h**) The normalized-gain magnitudes (**a**–**d**) and corresponding phases (**e**–**h**), each with respective box-and-whisker-plot summaries, using the same frequencies and methods described in Fig. 5. The ANOVA *p*-values are all insignificant in this figure.

The gains and phases at selected frequencies are shown for all animals in Fig. 7. The OHC-DC-junction data varied relatively little across animals, and the observations for the example animal (G637) generally held across all animals (Fig. 7).

### Motion along the basilar membrane

**Figure 8:**
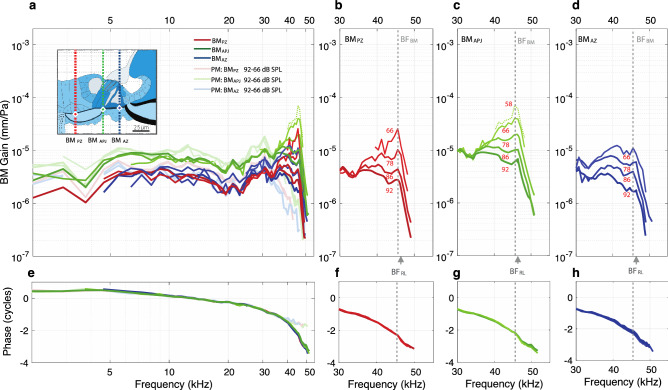
In vivo and PM motions across three BM locations (BM_PZ_, BM_APJ_, and BM_AZ_), for specimen G637. (**a**) In vivo (darker colors) and PM (faded colors) BM_PZ_, BM_APJ_, and BM_AZ_ gains with respect to the stimulus pressure (in units of mm/Pa). The inset drawing shows the measurement locations for BM_PZ_ (red diamond) BM_APJ_ (green diamond), and BM_AZ_ (blue diamond). Each colored vertical line indicates the lateral position and direction of the OCT A-scans. (**b–d**) The respective in vivo gains of the BM locations, showing active nonlinear amplification in the near-BF region. (**e–h**) The phase responses corresponding to panels (**a–d**). The green dotted line indicates the lowest-level BM_APJ_ measurement. All displacements are normalized by the sound pressure. The frequency axes in the first column are on a log scale, and those of the other columns are on a linear scale.

**Figure 9:**
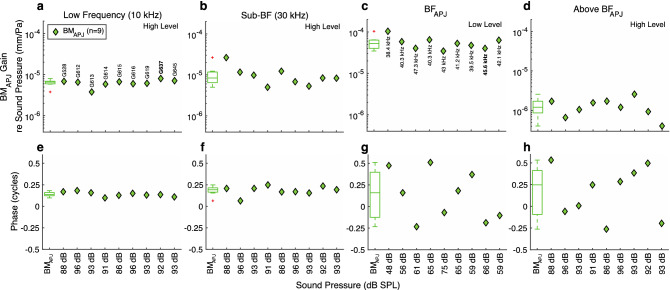
Summary comparisons across animals of the in vivo BM_APJ_ gains with respect to the sound pressure (in units of mm/Pa), for selected frequencies. (**a–h**) The gain magnitudes (**a–d**) and corresponding phases (**e–h**), each with a respective box-and-whisker-plot summary. The frequencies in the first two columns match those of Figs. 5 and 7, however those used in column 3 correspond to BF_BM_ (as shown in Fig. 8) instead of BF_RL_, and those used in column 4 are a quarter-octave above the frequencies used in column 3. In (**g**, **h**) phase was shifted up by 2 cycles from those shown in Fig. 8g.

The gains and phases in the example animal at three points along the width of the BM, i.e., in the arcuate zone (BM_AZ_), at the arcuate-pectinate junction of the BM (BM_APJ_), and in the pectinate zone (BM_PZ_), for in vivo and postmortem conditions, are shown in Fig. 8. The locations of BM_AZ_, BM_APJ_, and BM_PZ_ are shown in the inset of Fig. 8a. The gains and phases at selected frequencies are shown for all animals in Fig. 9.

As expected from previous work, the largest BM gain was at BM_APJ_, and the phase-versus-frequency response of the BM gain exhibited a phase delay that increased as frequency increased, consistent with excitation by a traveling wave. Near BF_BM_ the BM gain grew compressively, and below BF_BM_ the growth of the BM gain was mostly linear. Normalizing by the high-level BM_APJ_ gain shows that the motions at BM_AZ_ and BM_PZ_ were typically 6–10 dB lower than at BM_APJ_ (Supplementary Fig. 9).

## Discussion

Our measurements present a different and more complex picture of the motion within the OoC than has been conveyed by previously published measurements in gerbil. We were able to make more-detailed measurements because our OCT system has better spatial resolution than the systems used in previous papers^10,11,17–23^. In previous publications, the OCT images had comparatively lower resolutions, and although one could identify the BM as the first region of reflectivity though the round-window membrane (RWM) and scala tympani, other structures within the OoC could not be identified based on their shapes in the image. Instead, the locations of the tunnel of Corti and outer tunnel (ToC and OT, respectively, in Fig. 1g) were identified and the locations of structures such as the OHCs and/or the RL were inferred from the locations of the tunnels^18^. In contrast, our images are of sufficiently high quality to allow us to identify the RL and the bottoms (bases) of the OHCs (Fig. 1e) without needing to determine their positions from an overlaid standard image or from estimates of where reflectivity measurements originated from. In some cases, three reflectivity peaks could be seen on the RL at the tops of the three rows of OHCs (i.e., the end where the bundle of stereocilia protrudes). We identified measurements at different radial locations according to the OHC row they were closest to, but the alignment may not have been exact. For instance, we do not know if the structures that produce the reflectivity peaks on the RL correspond to an OHC top or an adjacent PhP top. Despite this uncertainty, our ability to discern radial positioning along the OHC rows is far greater than any previous publication, and this is the first publication that we are aware of that could definitively identify the RL in the gerbil basal region and measure from it.

### Our data compared to previous reports

Previous reports used a variety of optical-beam wavelengths, bandwidths, and motion-detection processing, and since the reflectivity of a structure depends on the wavelength, motions from different structures were likely to have been measured in different reports. In addition, the motion measured depends on the viewing angle, in part because different viewing angles can change the reflectivity of a structure, possibly due to birefringent properties of collagen fibrils in cochlear structures^24^. Further, if the OoC is not viewed perpendicular to the BM, then supposedly “transverse” measurements will also include radial and longitudinal components. For instance, Cooper et al.^23^ measured from the gerbil 20-kHz region as viewed through the RWM. To view the 20-kHz region through the RWM, the OCT beam must be pointed tangentially toward the apex (Fig. 1c), such that the motion measured along the axial direction of the beam will be a combination of transverse and longitudinal motion. By changing the angle of their beam, Cooper et al.^23^ concluded that much of what they measured was longitudinal motion. We developed a gentle surgical technique that produces little trauma and allowed us to measure in the 40–50-kHz BF region. Measurements in this region allowed us to use an OCT beam angle that was almost perpendicular to the BM when viewed through the RWM (Fig. 1c). Thus, our measurements are dominated by the transverse vector component of motion, with little contribution from radial or longitudinal components (more on this below).

Motion measurements from within the OoC in the gerbil cochlear base have been reported by three other groups^10,11,17–20,20,21,23^. Our measurements differ from previous reports in that: (1) we measured at three transverse locations, i.e., the BM, the OHC-DC-junction and the RL, whereas previous measurements were only at two locations, the BM and in the “OHC region”; and (2) we measured at several locations radially across the OoC at points aligned with the three rows of OHCs. There are no previous reported systematic measurements made radially across the three rows of OHCs. We first consider the measurements at different distances from the BM; later we consider the measurements at different radial locations. This and all previous reports found the classic pattern of BM motion: in the BF region the BM motion was amplified (relative to the dead animal) and had compressive growth. At frequencies 0.5 octaves or more below BF, the BM response was not amplified and grew linearly with stimulus level.

In the gerbil base, we found motions within the OoC that were substantially different from the motions previously reported^10,11,17–23^. The primary reasons for these differences appear to be that we measured from different structures than previous reports, and/or that we used different viewing angles, and/or that we measured with higher spatial resolution. We measured motion at the RL and OHC-DC-junction and identified those regions from images that were clear enough that we can be confident that those are the structures that were measured (Figs. 1, 3a). The images from the other reports did not allow such definitive identification of the structures producing the measured reflections, and the measured structures were referred to loosely as the “OHC region”^20–22^, the “hot-spot” in the OHC-DC region^23^, or as the “RL”^18^ (although “RL” in this case was a blind identification that has been interpreted as corresponding to the “OHC region”)^20^. We will refer to the non-BM measurements from the previous reports as “OHC region” measurements. A simplification that may be fairly accurate is that the “OHC region” motions reported by others show a combination of the motions we measured at the OHC-DC-junction and the RL.

For frequencies near BF, the previously reported “OHC region” motions showed compressive nonlinearity and more gain than the corresponding BM motion. We found compressive nonlinearity near BF in both the OHC-DC-junction and RL motions. Near BF, our RL motions look like the reported “OHC region” motions and usually had more gain than the corresponding BM motion (for RL_2_ and RL_3_, but not always for RL_1_ at the highest level; Fig. 5). In contrast, the OHC-DC-junction motions near BF were generally less than the corresponding BM motion (compare Figs. 6a and 9c), and had a substantially different pattern versus frequency than the reported “OHC region” motions.

For frequencies more than 0.5 octaves below BF (low frequencies), the previous reports found the “OHC region” motions to be substantially higher (10–20 dB) than the corresponding BM motion in the live animal, but similar to the corresponding BM motion in the dead animal, which shows that the “OHC region” motions received substantial amplification at low frequencies. Our low-frequency OHC-DC-junction motions were also 10–20 dB higher than the corresponding BM motion in the live animals, and similar to the corresponding BM motion in the dead animals. Thus, at low frequencies our OHC-DC-junction motions were similar to the previously reported “OHC region” motions. In contrast, at low frequencies our BM_APJ_-normalized RL gains were seldom very different from 0 dB (Figs. 4, 5a). Interestingly, across specimens the low-frequency motions of RL_1_ had phases similar to the corresponding BM_APJ_ motion (Fig. 4e and Fig. 5e), but RL_2_ and RL_3_ had phases that were advanced by about 0.25 cycles at the lowest frequencies (Fig. 5e). Our data show that at low frequencies the RL-gain magnitudes were similar to the corresponding BM_APJ_-gain magnitude (Fig. 5a), but that the OHC-DC-junction motions (which are of locations between the RL and BM) were 10–20 dB greater (Fig. 7a)!

### RL motion *above* BF

**Figure 10:**
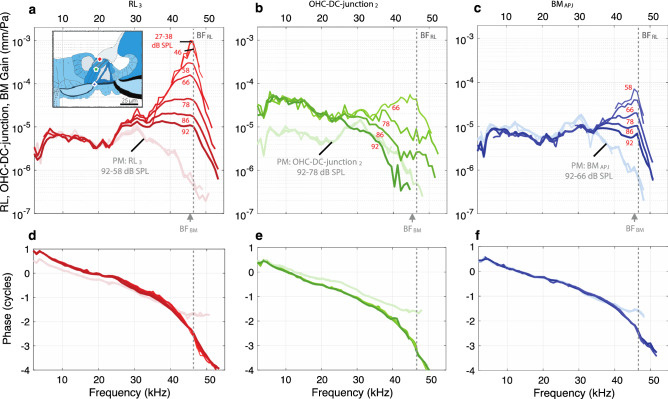
In vivo (darker colors) and PM (faded colors) gain comparisons across different OoC structures along a transverse direction, for specimen G637. (**a–c**) The respective gain magnitudes of RL_3_ (red), OHC-DC-junction_2_ (green), and BM_APJ_ (blue), all relative to the sound pressure (in units of mm/Pa). The inset drawing in panel (**a**) shows the measurement locations for RL_3_ (red circle), OHC-DC-junction_2_ (green pentagon), and BM_APJ_ (blue diamond). (**d–f**) The phase responses corresponding to (**a–c**). The available stimulus levels vary across the structures due to different signal-to-noise ratios.

One interesting finding is that at frequencies above BF_RL_, the RL gain was larger than the corresponding BM gain (Figs. 4, 5; Supplementary Figs. 2–4). This was due to the BM having a slightly lower BF than the RL, and to the BM response falling very sharply as frequency increased, starting at frequencies just above BF_BM_, whereas the RL motion did not fall as sharply (Fig. 10, a versus c). Similar findings were made in gerbils^10^ and guinea pigs^6^. One explanation for this is that traveling-wave cochlear amplification falls rapidly above BF_RL_, which decreases BM motion rapidly. RL motion rides on top of the BM motion and also decreases, but the RL motion above BF also receives local amplification by the OHCs, just as it does below BF_RL_. Since the OHC stereocilia are no longer saturated by the drive from BM motion, they can amplify RL motion more than at BF. This is consistent with the effects seen by systemic injection of salicylate or furosemide that alters cochlear function^21,22^.

### Motion at different radial positions within the organ of Corti

This is the first report of in vivo motion within the OoC across the three OHC rows, either at the top or the bottom of the OHCs. For frequencies near BF_RL_, RL_3_ had significantly more motion (10 ± 1 dB) than RL_1_ (Fig. 5c), but at the OHC-DC-junction there was relatively little difference across the three OHC rows (Fig. 7c). For frequencies below BF, the RL-gain magnitudes were similar across the three rows but the phase of RL_1_ was about 0.25 cycles lower than those of RL_2_ and RL_3_ (p < 0.006; Fig. 5a), while for the OHC-DC-junction the gain magnitudes and phases were similar across the three rows (Fig. 7a). These observations are illustrated in a four-panel movie (Supplementary Movie 1) showing in vivo motions at 9.8 kHz and the 46.4-kHz in vivo BF (at 86 and 66 dB SPL, respectively; Supplementary Movie 1A, B) and postmortem motions at 9.8 kHz and the 29.9-kHz postmortem BF (~ 0.5 octaves below the in vivo BF; both at 86 dB SPL; Supplementary Movie 1C, D). Note that different magnification factors were applied to the motions across the panels A–D. The fact that the in vivo RL_1_ and RL_3_ motions differ significantly from one another near BF whereas the OHC-DC-junction_1_ and OHC-DC-junction_3_ motions remain similar (see the Supplementary Movie 1B) seems to imply that the RL mosaic comprised of OHC and PhP cuticular plates and adhesion molecules is much more flexible than the DCs connected to the OHCs at their bottom surfaces. Postmortem, the measured points moved together approximately in phase at both the low frequency (Supplementary Movie 1C) and near the postmortem BF (Supplementary Movie 1D), consistent with the ter Kuile model that assumes the RL moves as a stiff plate^25^.

Two previous reports provide sparse measurements from live animals that are consistent with our observation of more motion at RL_3_ than at RL_1_. Fallah et al.^11^ reported measurements done at two radially separated “OHC regions” and found more motion in the more-lateral location in one case, but not in the other. Dewey et al.^16^ reported one set of interpolated measurements along the top of the mouse OoC in the apical region and found similar motions at RL_1_ and RL_3_ with 5 dB greater motion in the middle near RL_2_. Additional measurements for comparison include the BM radial-profile motions in guinea pig^26^ and gerbil^23,27^. The guinea-pig radial motion was reported to be “bi-lobed”, with the highest motions below the tunnel of Corti and below DC_1_. In contrast, the gerbil radial profile is simpler, with a single peak near BM_APJ_^27^. This simpler motion in gerbil was attributed to the arch in BM_PZ_, which effectively makes it a stiff plate^28^. The hinging rotations of the inner and outer pillar cells and BM_AZ_ also make BM_PZ_ appear as a plate. The net effect of this is two stiff plates rotating about the spiral limbus or OSL at their far ends that join together at BM_APJ_ to produce maximum motion at that location. Thus, the radial profile of the gerbil BM is more platelike, whereas the radial profile of the gerbil RL is not like that of a plate but rather bends and possibly stretches (Supplementary Movie 1). Also relevant are measurements with electrical stimulation in an excised gerbil middle-turn preparation that found differences in motion in the radial and longitudinal directions across the three OHC rows at the bottoms of the OHCs^29^.

What do the differences in the motions across RL_1_, RL_2_, and RL_3_ tell us about what is happening in the cochlea? First, we must acknowledge that our one-dimensional displacement measurements are close to the direction that is transverse (orthogonal) to the plane of the BM, which provides a global OoC coordinate system, but we do not have a measure of how close. Second, the motion of each OHC takes place along its own long axis, which is positioned at an angle relative to the transverse axis of the BM. In addition, this angulation of each OHC long axis relative to the BM transverse axis varies across the three OHC rows^3^ (Fig. 1g and Supplementary Movie 1). The RL and OHC-DC-junction motion measurements were therefore not taken along the long axes of the OHCs, and thus they represent projections of the axial OHC motions of each OHC row onto the measurement direction^30^. Across animals, the motions of RL_2_ and RL_3_ were similar and those of RL_1_ were different (Figs. 3, 4 and 5). At high frequencies RL_2_ and RL_3_ had higher gains than RL_1_, but all had similar phases, while at low frequencies they all typically had similar gains but RL_2_ and RL_3_ had ~ 0.25-cycle phase advances (or equivalently 0.75-cycle phase lags) relative to RL_1_. There are several (not mutually exclusive) classes of interpretations for the motion differences: (1) the RL motions are transverse motions with little contribution from radial and/or longitudinal RL motion, and (2) the differences are primarily due to non-transverse (radial and/or longitudinal) components of the local motions being projected onto our nearly transverse measurement direction, with (2a) individual OHC-row motions that were aligned to the local OHC long axis, or (2b) non-transverse motions that are not related to the local OHC long axis. One simple way for there to be more transverse motion at RL_3_ than RL_1_ is for these motions to be made up of a transverse OoC motion plus a transverse rotation of the RL about a fulcrum near RL_1_ (e.g., at the top of the pillar cells as suggested by Nowotny and Gummer^31,32^ and as shown in Supplementary Movie 1). Our RL data might be matched if the rotation and translation were in-phase near BF, and if the rotation had a phase advance at low frequencies. Counting against the RL-rotation hypothesis is its implication that the RL_2_ motion should be about half-way between the RL_1_ and RL_3_ motions, whereas the RL_2_ motion is instead similar to the RL_3_ motion. However, there are not enough RL_2_ measurements to rule out the rotation hypothesis. If RL rotation is not a big factor, and the motion is all transverse, this would mean that there is significant deformation of the RL.

A possibility is that the RL_3_ and RL_2_ motions are greater than those of RL_1_ because of non-transverse (radial and/or longitudinal) motions. This might explain the differences observed at low frequencies, because a phase difference of ~ 0.25 cycles can come from combining motions that are occurring in two perpendicular directions^23^. Also, the motions at RL_1_ and BM_APJ_ can be expected to be similar because these two regions are connected by the relatively stiff outer pillar cells (see Supplementary Fig. 10). However, near BF the RL_3_/RL_1_ ratio is about 10 dB or a factor of 3. To account for this large difference in motion there would have to be over an order-of-magnitude more non-transverse motion than transverse motion at RL_3_ compared to RL_1_, because the viewing angle is nearly transverse and would record only a small fraction of any radial or longitudinal motion. Non-transverse motion that is more than an order of magnitude larger than transverse motion in RL_3_, but not in RL_1_, seems unlikely. This does not mean that the RL_2_ and RL_3_ measurements do not include any non-transverse contributions, but that non-transverse contributions seem unlikely to account for the observed differences between RL_3_ and RL_1_. One conclusion from all of this is that to account for our measurements, it is likely that there is substantially more transverse motion of RL_3_ compared to RL_1_, which means that there must be substantial deformation of the RL. In the mouse, Dewey et al.^16^ reported finding a reversal of the motion along the top of the OoC in the region next to RL_3_ at the attachment of the PhP furthest from the pillar cells, which would be consistent with this part of the RL being not very stiff.

One implication of the interpretation that the increased motions of RL_2_ and RL_3_ compared to RL_1_ are due to transverse motion, is that the OHCs produced more motion at RL_2_ and RL_3_ than at RL_1_. This could be because all OHC rows receive the same deflections to their stereocilia and produce the same OHC force, but that RL_1_ motion is restrained by its nearby attachment to the pillar cells (see Supplementary Fig. 10). Alternately, the OHCs of rows 2 and 3 might instead receive larger stereocilia deflections and therefore produce more force. This would be consistent with there being more radial motion at RL_2_ and RL_3_ that deflects the OHC stereocilia, although such radial motion might contribute little to the transverse motion that we measured. Mouse OoC cytoarchitecture measurements show differences in the radial and longitudinal angles between the three rows of OHCs and their PhPs^3^. If these differences in cytoarchitecture hold in the gerbil, this could produce different radial and longitudinal motion components across rows. Recently it was hypothesized that this increased radial motion could originate from expansion or contraction of the outer tunnel, whose upper wall connects to the RL near RL_3_ via the tectal-cell extension^33^. Measurements from two or more angles are needed to decompose the motion directions into vector components at RL_1–3_ to test these possibilities.

Yet another interpretation of the radial changes in transverse RL motion we report pertains to the relative impedances at the tops and bottoms of the OHCs. The OHC_1_ row is adjacent to the outer pillar cells, located between the pillar heads and the BM, which are relatively stiff compared to other nearby structures. OHC_3_ and OHC_2_ are further away from the pillar cells, situated among DCs and PhPs, which are expected to be less stiff than the pillar cells. The factor of 3–4 difference in motion between RL_3_ and RL_2_ vs. RL_1_ near BF_RL_ could imply that there is a similar difference in the local impedance between the outer-pillar-cell-dominated OHC_1_ row and the less-constrained OHC_3_ and OHC_2_ rows (Supplementary Movie 1B). However, this expected difference in impedance across rows does not apparently manifest in the same way at lower frequencies (Supplementary Movie 1A), which argues against the radial variations in transverse RL motion being explained entirely by impedance differences. Much work is needed to understand these complicated interactions with frequency, level, and OoC location.

One approach to better understand these different possibilities would be to use a finite-element model of the RL mosaic that incorporates the radial and longitudinal angles of the PhPs across the three OHC rows, as well as the outer-tunnel fluid space, and that includes frequency-dependent impedances. Regardless of the underlying reason(s) for the observed motion differences, these measurements suggest that the RL does not move as a stiff plate hinging around the pillar-cell heads as has been assumed for over a century^25,34,35^, but that its mosaic-like structure may instead bend and/or stretch. Understanding the specifics of RL motion is fundamental to understanding both cochlear amplification via OHC stimulation and sound transduction via inner-hair-cell stimulation.

### Implications for feedback and cochlear amplification

It is well-established that amplification within the cochlea consists of a feedback loop comprised of forward transduction (from BM mechanical motion to rotation of the pillar cells, shearing between the TM and RL, motion of the OHC stereocilia bundles, and establishment of the OHC receptor potential) and reverse transduction (from OHC receptor potential to OHC axial-force generation, and motion of the BM and RL) pathways^26,36,37^. Central to this process is the tilting and rotation of the RL as the BM moves, thus further amplifying the shear displacement between the TM and RL and hence amplifying the angular rotation of the OHC hair bundles^26^.

The phasing of OHC motion to amplify BM motion in the feedback loop arises from a change in the phase of RL motion that, as frequency is increased, begins rather abruptly at about 0.5 octaves below BF^9,12,13^. Since the Dong and Olson^12^ measurements that identified this rapid phase change were done in the base of the gerbil cochlea, it seemed possible that a motion correlate of this transition would become evident in our measured motions. At frequencies less than about 0.5 octaves below BF (where there is no traveling-wave amplification) the OHC top moved *less* than the OHC bottom (Fig. 10a), but at frequencies above this (where there is traveling-wave amplification) the OHC top moved *more* than the OHC bottom (Fig. 10b). One interpretation from a feedback perspective is that the OHCs were working in a negative-feedback fashion at frequencies 0.5 octaves or more below BF_RL_, but at higher frequencies the OHCs were working in a positive-feedback fashion that produced traveling-wave amplification^38^.

## Methods

### Animal preparation

Gerbils have the advantages of being small and of having been used in previous experiments involving intra-cochlear pressure^39–41^ measurements, OCT-based cochlear measurements^11,20–23,42^, and cochlear measurements using other optical techniques^17,18,43–45^.

In this study, healthy female Mongolian gerbils, (N = 19, aged 5–11 weeks, weight range 41–76 g) were provided by one supplier (Charles River Laboratories). All gerbils arrived at the Massachusetts Eye and Ear (MEE) animal care facility at an age of about 4 weeks. Upon arrival, the animals were housed in same-gender groups of three gerbils per cage. Thereafter, gerbils were quarantined for a period of 1 week. In vivo OCT vibrometry results are reported for the nine left ears which had good cochlear sensitivity, as assessed with distortion-product otoacoustic emission (DPOAE) criteria (see Supplementary Note 1). Approximately the same surgical procedures and measurement methods were used to collect the reported results.

To minimize cochlear pathology, care was taken throughout to reduce noise- and vibration-related trauma^46^. A key surgical technique was opening the bulla after applying phosphoric acid gel (PAG, PULPDENT Corporation, MA, USA) to thin (decalcify) and soften the bone^47^. PAG was applied for 10 s and then wiped off using a micro-wipe tip. After repeating this procedure three times, a narrow opening in the posterolateral wall of the bulla was made with fine forceps. The tympanic membrane, malleus, incus, stapes, and RWM were all kept intact.

Surgical procedures and anesthesia protocols were described previously^33^. The initial anesthesia was induced by an intraperitoneal (IP) injection of sodium pentobarbital (70 mg/kg), followed by a subcutaneous (SQ) injection of acepromazine (1 mg/kg) mixed with atropine (0.06 mg/kg). Lidocaine (1%) with epinephrine (1:100,000) was applied topically to the skin over the top of the skull and around the left ear. To maintain adequate anesthesia, 1/3 of the initial dose of sodium pentobarbital was given approximately every 45–60 min, and the depth of anesthesia was evaluated every 30–60 min via toe-pinch response and/or an increase of more than 10% in the monitored heart rate. Atropine (the full initial dose) was reinjected after 2–3 h in case of respiratory difficulty. After the in vivo measurements, an intraperitoneal injection of Fatal Plus (> 150 mg/kg) produced euthanasia. Within 5–10 min after the injection, the heart typically stopped and the animal stopped breathing. The postmortem vibration measurements were done 5–60 min after the animal stopped breathing and had no heartbeat. This study was approved by the Institutional Animal Care and Use Committee (IACUC) at MEE. All methods and procedures were performed in accordance with the approved MEE protocol and written up following the ARRIVE (Animal Research: Reporting of the In Vivo Experiments) guidelines.

### Stimulus generation and acoustic measurements

For signal generation and sound-pressure control, we used a National Instruments (NI) PXI-4461 dynamic signal acquisition board mounted in an NI PXI-1031 chassis with an NI PXI-8196 embedded computer (NI, TX, USA) at a sample rate of 192 kHz, which is just below the board’s maximum rate of 204.8 kHz. For measuring DPOAEs, this system was controlled using the LabVIEW-based Cochlear Function Test Suite (EPL_CFTS, version 2.37-R3041)^48^. For the OCT vibrometry measurements, we used custom stimulus-generation and synchronous-measurement software (SyncAv, version 0.42)^49^, which generated a sequence of pure tones (2–63 kHz, ~ 0.8-kHz linear frequency steps) and a trigger signal to control OCT acquisition. The board output was amplified by a Techron Model 5507 power amplifier (AE Techron, IN, USA), which drove a Parts Express 275-010 tweeter speaker mounted in a custom-built closed-field acoustic assembly. During the experiments, ear-canal pressure was measured by a calibrated Knowles FG-23329 electret microphone and probe tube, with the probe-tube opening placed 1–2 mm from the tympanic membrane. Initially, an in situ pressure response with a constant stimulus voltage as a function of frequency was measured. The measured microphone response was then used to vary the stimulus voltage to equalize the ear-canal pressure and produce a nearly flat response across frequencies (Fig. 2a). This equalization voltage curve was scaled in approximately 10-dB steps (or ~ 5-dB steps above 85 dB SPL) to produce the range of stimulus levels measured in the experiment. The value for the stimulus sound pressure level (SPL; in dB) associated with each measurement was calculated as the average measured pressure across frequencies (0 dB SPL = 20 µPa). The stimuli were varied from ~ 30 to ~ 90 dB SPL across experiments, and the actual SPLs varied ± 4 dB due to small differences in the position of the acoustic assembly in the ear canal from the initial equalization steps.

### Monitoring cochlear sensitivity

Cochlear sensitivity was monitored by DPOAEs measured before and after surgery, and approximately every 20 min during in vivo measurements using the EPL_CFTS software (Supplementary Fig. 1)^48^. DPOAEs were evoked by a series of tones at frequencies f1 and f2 (f2/f1 = 1.2), with f2 varied from 2 to 63 kHz in octave steps below 30 kHz, and 2-kHz steps above 30 kHz. The two tones were presented with the level of the f2 primary 10 dB less than the f1 level in two separate runs at 50 or 70 dB SPL f1 primary levels. Across animals, the DPOAE amplitudes mostly ranged from ~ 15 to 25 dB SPL for f2 frequencies above 30 kHz and the noise floor was typically less than 0 dB SPL (Supplementary Fig. 1a). A cochlear region’s sensitivity was assessed from its DPOAE with f2 near BF, and was deemed adequately sensitive if the DPOAEs from 50 dB SPL primary tones were greater than 10 dB SPL (Supplementary Fig. 1b).

### OCT imaging and vibrometry

All OCT imaging and vibrometry measurements were made using an SD-OCT system with a 900-nm center wavelength and a high-speed (up to 248-kHz) line-scan camera (GAN620C1, Thorlabs, Germany). The OCT system combined two near-infrared superluminescent-diode light sources with center wavelengths of 847.5 nm and 929.6 nm, with full width at half maximum (FWHM) spectral bandwidths of 63.4 and 95.8 nm, respectively. Before performing the fast Fourier transform (FFT), the SD-OCT system multiplied the recorded interference spectrum with an apodization (Hann) window function, which caused a broadening of the axial beam profile by a factor of 2 and had an impact on the axial resolution. The penetration depth was ~ 1.44 mm (in water, with a refractive index of 1.33). The axial resolution was ~ 2.23 µm (in water) and the lateral resolution was ~ 8 µm, using a 36-mm, 0.055-NA, 2× objective lens (OCT-LK3-BB, Thorlabs, Germany). The lateral resolution of 8 µm is slightly greater than the 6 µm diameter of OHCs but is approximately equal to their intercellular distances as measured in mice^3^.

The OCT measurements were made with custom LabVIEW (NI, TX, USA)-based VibOCT software (version 2.1.4), built using the Thorlabs SpectralRadar software development kit (version 5.4.8). This system can provide (1) real-time video images that were used in the anatomical approach to determine the region of interest for OCT scans; (2) depth-resolved 1D A-scans (reflectivity vs. axial depth, e.g., Fig. 1d); (3) 2D cross-sectional B-scans (axial depth vs. scan range, e.g., Fig. 1e) with software-selectable x–y offset and scan rotation angle; (4) 3D volumetric C-scans (reflectivity vs. axial depth with a scan range over two perpendicular axes), and (5) vibrometry data-acquisition measurements, at a single point or for an entire A-line (axial depth), in terms of the displacement and phase synchronized to tones (or other stimuli).

OCT vibrometry was synchronized by an external trigger provided by the SyncAv software that generated the tones. The OCT camera required two trigger pulses within a tone cycle. This limited the trigger pulse generated by SyncAv to frequencies below 48 kHz. To overcome this limitation, we used a frequency-doubling program running on a PXI-6221 board (NI, TX, USA) to double the trigger-pulse frequency, which allowed OCT vibrometry measurements at frequencies up to 96 kHz. The performance of the OCT hardware and VibOCT software were evaluated by measuring the displacement and phase of a small piezoelectric vibrator over a wide range of frequencies and stimulus levels. The OCT displacement and phase readings were then compared to measurements made using a calibrated laser-Doppler vibrometer (LDV; Polytec OFV501/OFV2600, Irvine, CA). Phase differences between the two were compensated for using custom post-processing software written in MATLAB (R2020a; Natick, MA, USA). The difference between LDV and OCT vibration measurements were typically within 1 dB (but up to 2 dB at some frequencies) in magnitude and 0 degrees in phase (by definition). With this setup on the bench, the system noise floor was ~ 35 pm at 0.1 kHz and decreased to below 20 pm from ~ 20 kHz up to 63 kHz.

The animal was placed on a two-stage goniometer (07-GON-503, Melles Griot, Carlsbad, CA, USA) that was positioned on top of a 3-axis micro-manipulator (OCT-XYR1, Thorlabs, Germany) mounted on a vibration-isolation table. The head was oriented so that measurements could be made through the intact RWM with the viewing angle adjusted to be close to transverse with respect to the OoC and BM. This viewing angle provided access to BFs in the 40–50 kHz range (see Fig. 1c). Oriented using the real-time OCT images, we chose a region of interest (e.g., BM, OHC-DC-junction, RL) from an A-scan depth profile (Fig. 1d). We then made measurement scans (M-scans), which consist of A-scan depth profiles as functions of time that contain the vibration information. Each B-scan (for imaging) and M-scan (for vibrometry) had 8,192 samples. A second FFT was performed along the time axis of the interferometric phase data for the extraction of vibration displacement and phase^50,51^. The vibration responses to a series of sequential tones were saved to allow further viewing and post-processing at any A-scan location. With our custom software, the data could be analyzed during the experiment within minutes of being acquired.

### Statistics

To determine the significance of the in vivo motion (magnitude and phase) of each structure (e.g.*,* RL_3, 2, 1_, OHC-DC-junction_3, 2, 1_), we performed multiway (*n*-way) analysis of variance (ANOVA) calculations for testing the effects of multiple factors using the built-in MATLAB function anovan (MATLAB R2020a; Natick, MA, USA). The analyses indicated that the different locations were statistically independent (p < 0.05 criterion). The statistical results are shown in Fig. 5 (RL) and Fig. 7 (OHC-DC-junction).

## Supplementary Information

Supplementary Information 1.

Supplementary Video 1.

## Data Availability

All data needed to evaluate the conclusions in the paper are present in the paper. Additional data related to this paper may be requested from the author Sunil Puria, PhD.
