# Supplementary material for: Cochlear motion across the reticular lamina implies that it is not a stiff plate
Source: Sci Rep. 2022 Nov 4;12:18715. doi: 10.1038/s41598-022-23525-x (PMC9636238; doi:10.1038/s41598-022-23525-x)
Supplement: Supplementary file 1 — Supplementary Information 1. [file 41598_2022_23525_MOESM1_ESM.pdf]

Supplementary Information (SI)

Cochlear motion across the reticular lamina implies that it is not a stiff plate

Nam Hyun Cho [1,2] and Sunil Puria [1,2,3,*]

Affiliations
[1]Department of Otolaryngology–Head and Neck Surgery, Harvard Medical School, Boston, MA, 02114, USA
[2]Eaton-Peabody Laboratories, Massachusetts Eye and Ear, Boston, MA, 02114, USA
[3]Speech and Hearing Bioscience and Technology Program, Harvard University, Cambridge, MA, 02138, USA

* Correspondence: Sunil_Puria@meei.harvard.edu

File name: "Supplementary Movie 1.mp4". Animations of in vivo (A, B) and postmortem (C, D) motions at selected frequencies (indicated in the bottom-left corner of each panel) and sound pressure levels (indicated in the title of each panel) from a representative animal (G637). The top row shows in vivo motions at a low frequency near 10 kHz (A) and at the in vivo best frequency (BF) of the RL (B). The bottom row shows postmortem motions at the same low frequency (C) and at the postmortem BF (~½ octave below the in vivo BF; D). Each panel shows the same background OCT cross-sectional image along with the vibrometry measurement points ("+" symbols) and magnified animations of the displacements of those points (colored "o" symbols). The points displayed are (from left to right and top to bottom): $RL_{3,2,1}$ (red, green, and blue symbols), PC_H (the head of the pillar cells; purple symbol; only for in vivo), OHC-DC-$J_{3,2,1}$ (red, green, and blue symbols), and $BM_{PZ, APJ, AZ}$ (red, green, and blue symbols). The motions in each panel were magnified by a different scale factor (indicated in the upper-right corner) to emphasize the relative motions. Panel B is at a different sound pressure level (66 dB SPL) to show motions at the lowest level available for the in vivo BF.

Supplementary Note 1. Cochlea sensitivity was monitored using distortion product otoacoustic emissions (DPOAEs) at approximately 20-min intervals during in vivo OCT vibrometry measurements. Following the injection of Fatal Plus, Postmortem DPOAEs were collected after the animal stopped breathing and had no heartbeat, at which point DPOAE amplitudes dropped to the noise floor level (data not shown). Supplementary Figure 1a shows DPOAEs responses from across animals (n=9) evoked by two simultaneous tones at frequencies f1 and f2 (f2/f1 = 1.2), with f2 swept from 2 to 63 kHz in octave steps below 30 kHz, and 2-kHz steps above 30 kHz. The two tones were presented with the f2 level 10 dB less than the f1 level, with the f2 level at 50 and 70 dB SPL in separate runs. A cochlea was considered to be healthy if the 2f1-f2 DP amplitudes from 50 dB SPL f1 tones were greater than a 10 dB SPL criterion.

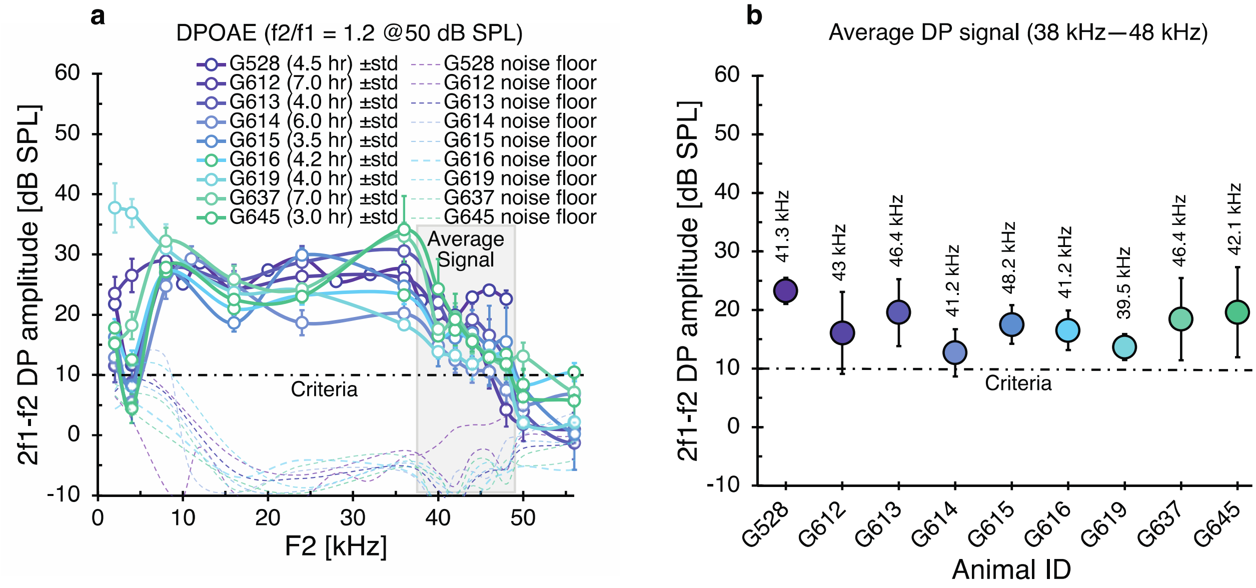

Supplementary Figure 1. Cochlea sensitivity monitored using DPOAEs across animals (n=9). **a:** DPOAE amplitudes and noise floors measured at 2f1-f2, during in vivo OCT vibrometry. The symbols and solid lines represent the means and standard deviations of the DPOAE amplitudes, and the dashed lines represent the averaged noise floors, measured during the in vivo experiments (the durations of in vivo measurement times are shown in the legend). **b:** Average DP amplitudes near the best frequency (BF) frequency region, i.e., 38 kHz–48 kHz (gray box in **a**). The X axis is animal ID, sorted by animal numbers as in the main manuscript figures. Each animal's BF is labeled above its symbol. The black dashed horizontal line represents the 2f1-f2 DPOAE amplitude criterion of 10 dB SPL. All animals passed this criterion.

Supplementary Note 2. Examples of reticular-lamina (RL) gain at three radial locations. The three radial locations (RL_3, RL_2, RL_1) correspond to the apical surfaces of the three outer-hair-cell (OHC) rows. Shown are data from three animals including the one in the main manuscript (G637). The gains and phases from the living (dark colors) and postmortem (PM; faded colors) animals were normalized by the averaged BM_{APJ} gain from the corresponding animal, measured at a high level to remove the contribution of the BM traveling wave to RL motion. BM gains were measured at the junction of the arcuate zone (AZ) and pectinate zone (PZ), BM_{APJ}

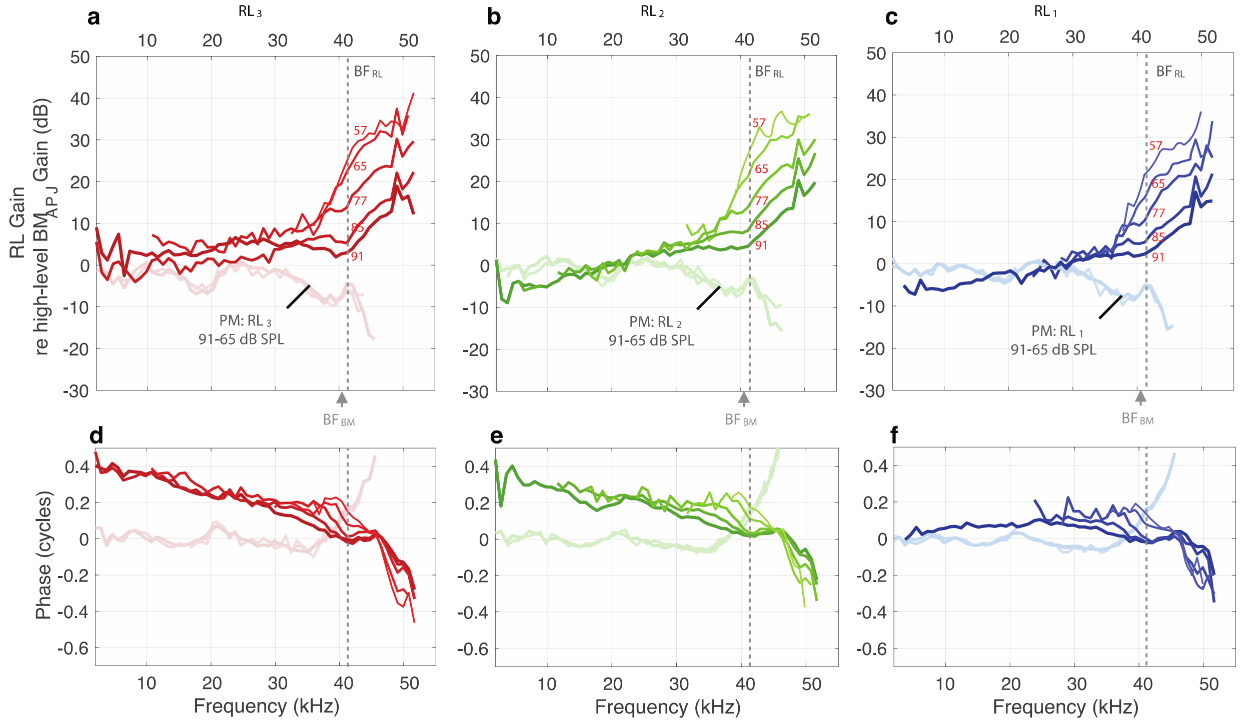

Supplementary Figure 2. Gerbil G614: **a–c:** In vivo and PM gains for RL_3, RL_2, and RL_1, respectively, normalized by the high-level BM_{APJ} gain (in dB). **d–f:** The phase responses corresponding to panels **a–c**. The baseline high-level BM_{APJ} gain used for normalization was calculated as the average of five measurements made at 91 dB SPL.

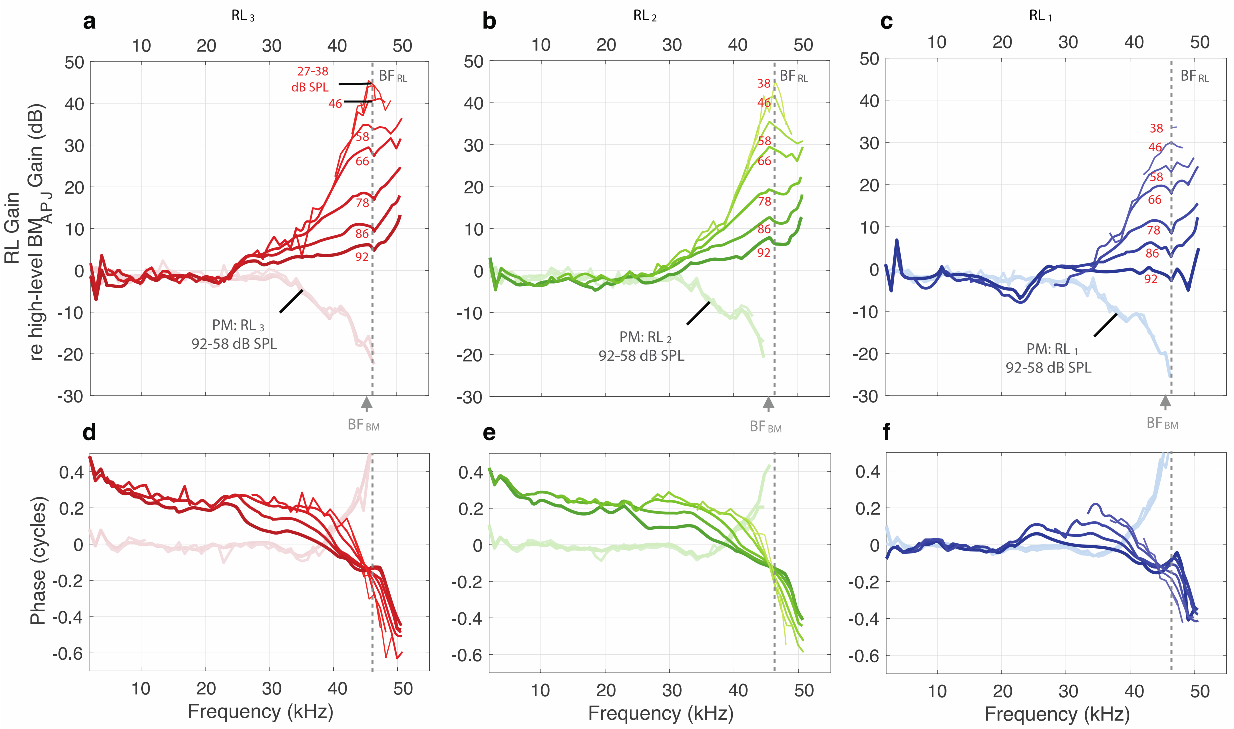

Supplementary Figure 3. Gerbil G637: **a–c:** In vivo and PM gains for RL_3, RL_2, and RL_1, respectively, normalized by the high-level BM_{APJ} gain (in dB). **d–f:** The phase responses corresponding to panels **a–c**. The baseline high-level BM_{APJ} gain used for normalization was calculated as the average of ten measurements made at 92 dB SPL. These data are also shown in Fig. 4 of the main manuscript.

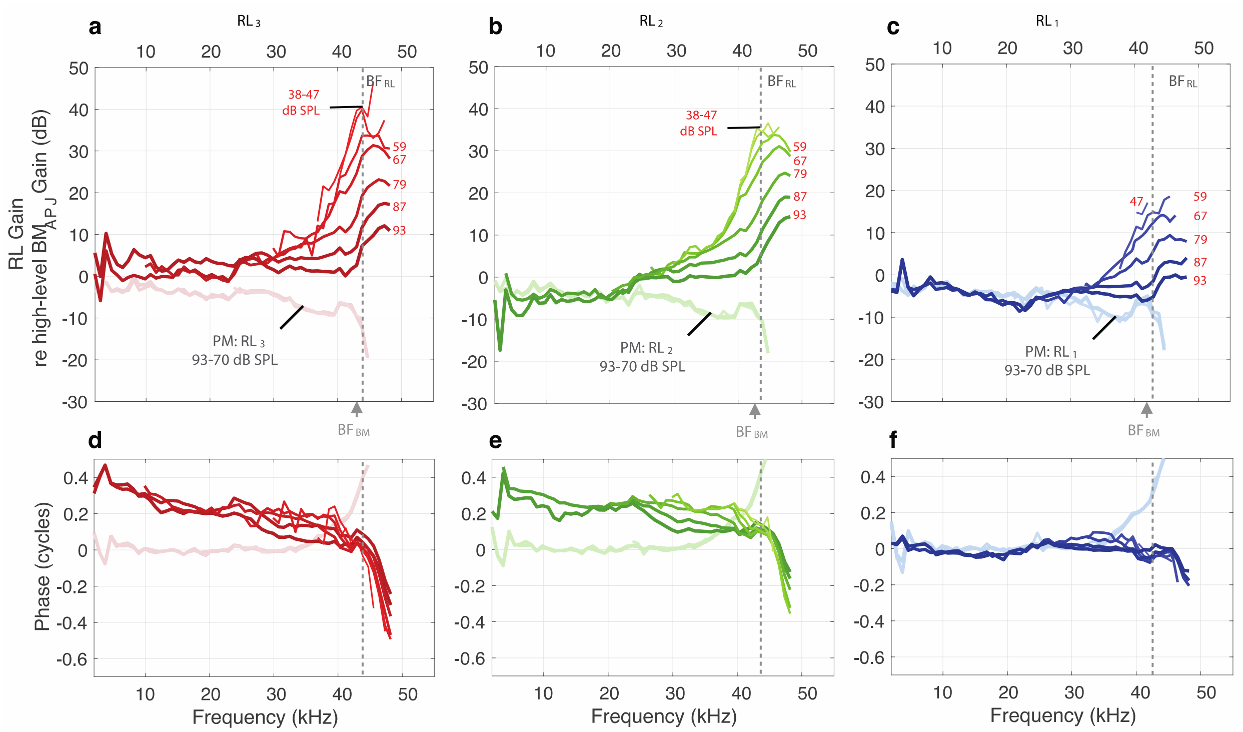

Supplementary Figure 4. Gerbil G645: **a–c:** In vivo and PM gains for RL_3, RL_2, and RL_1, respectively, normalized by the high-level BM_{APJ} gain (in dB). **d–f:** The phase responses corresponding to panels **a–c**. The baseline high-level BM_{APJ} gain used for normalization was calculated as the average of four measurements made at 93 dB SPL.

Supplementary Note 3. Supplementary Figures 5–8 show gains at three transverse locations from four animals: The locations are: RL_3, the junction between OHC row and its Deiters' cell (OHC-DC-junction), and BM_{APJ}. These figures are similar to Fig. 10 in the main manuscript.

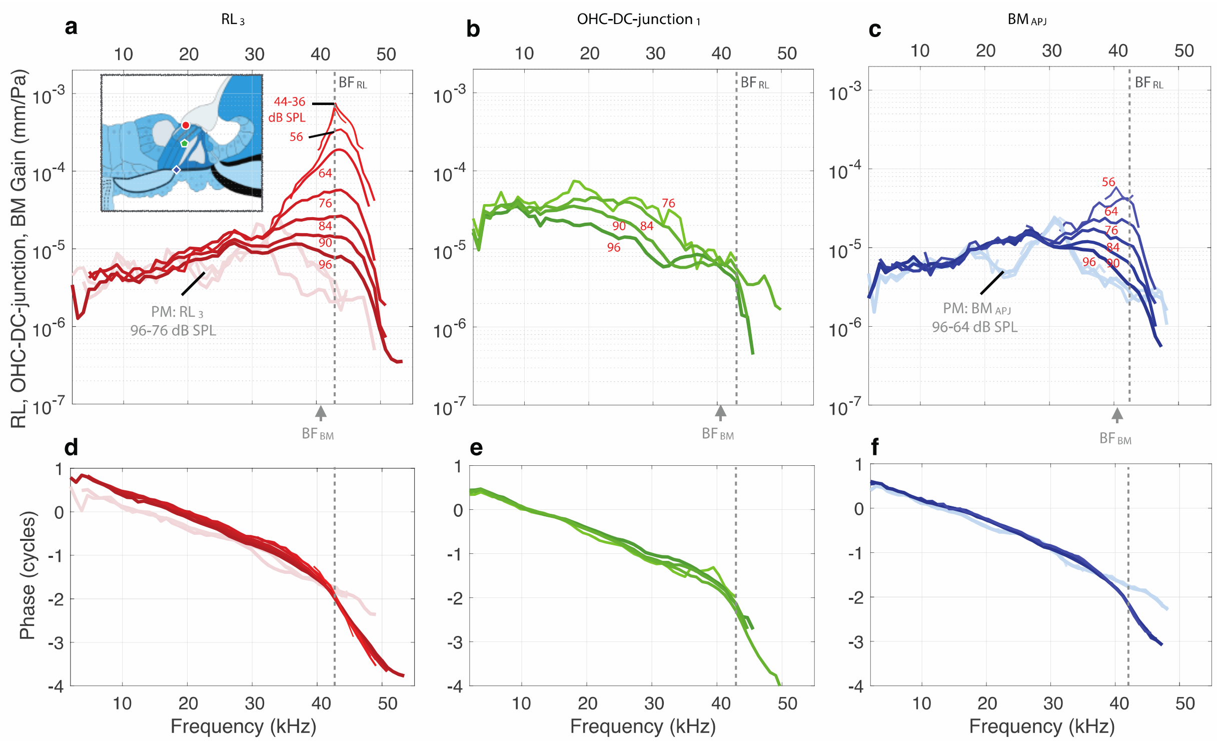

Supplementary Figure 5. Gerbil G612: **a–c:** In vivo (darker colors) and PM (faded colors) gains for RL_3 (red), OHC-DC-junction$_1$ (green), and BM_{APJ} (blue), all relative to the sound pressure (units of mm/Pa). The inset drawing shows the measurement locations for RL_3 (red circle), OHC-DC-junction$_1$ (green pentagon), and BM_{APJ} (blue diamond). **d–f:** The phase responses corresponding to **a–c**. Note that the available stimulus levels vary across the structures due to different signal-to-noise ratios. No PM OHC-DC-junction$_1$ measurements were available for this specimen.

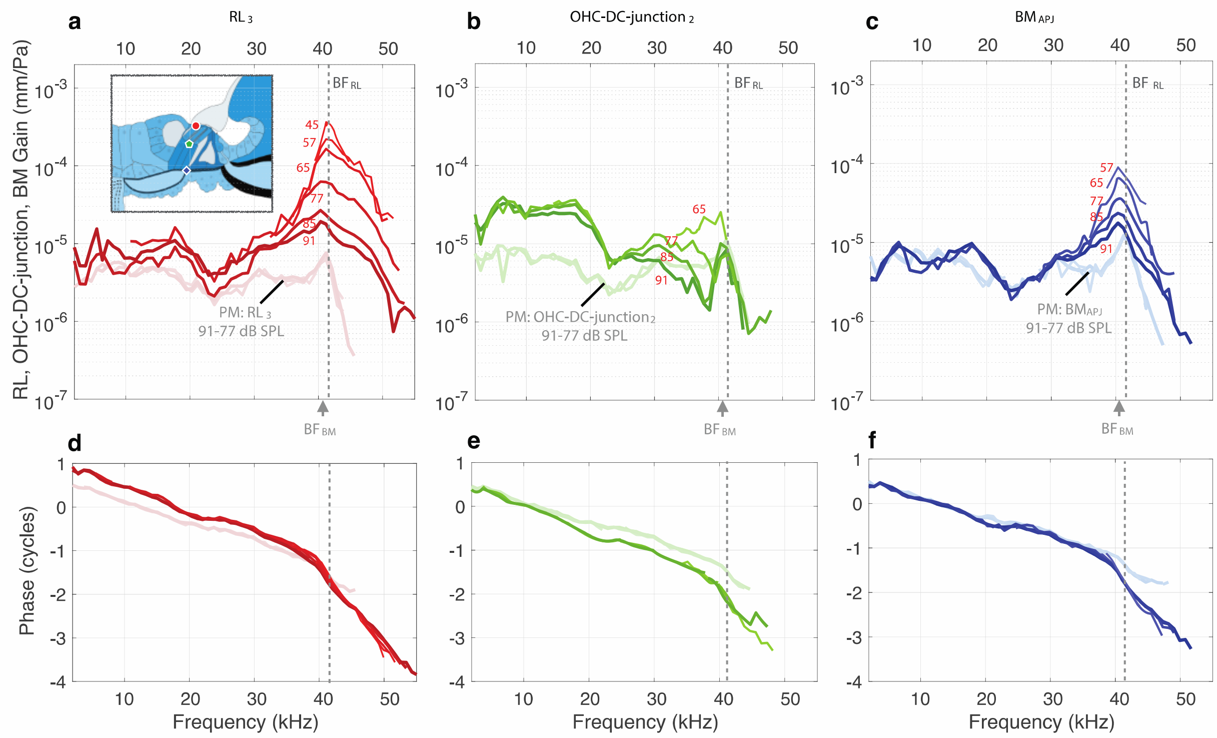

Supplementary Figure 6. Gerbil G614: **a–c:** In vivo (darker colors) and PM (faded colors) gains for RL_3 (red), OHC-DC-junction$_1$ (green), and BM_{APJ} (blue), all relative to the sound pressure (units of mm/Pa). The inset drawing shows the measurement locations for RL_3 (red circle), OHC-DC-junction$_1$ (green pentagon), and BM_{APJ} (blue diamond). **d–f:** The phase responses corresponding to **a–c**. Note that the available stimulus levels vary across the structures due to different signal-to-noise ratios.

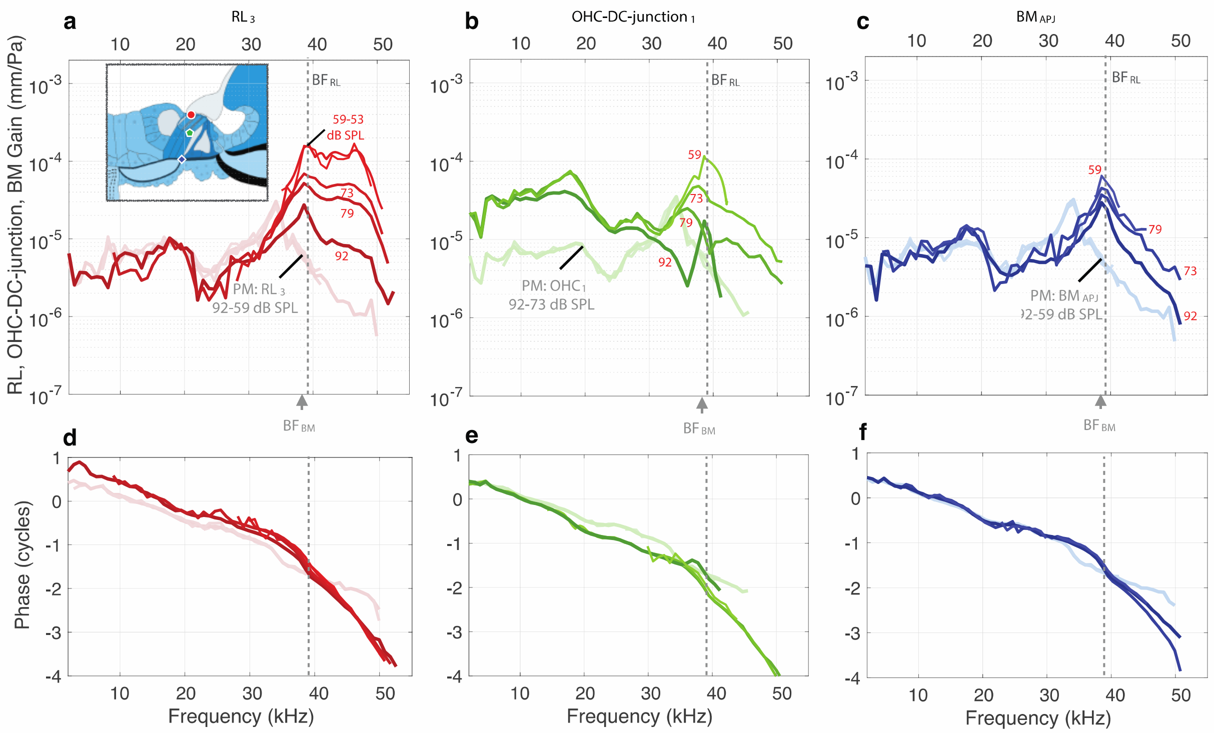

Supplementary Figure 7. Gerbil G619: **a–c** In vivo (darker colors) and PM (faded colors) gains for RL_3 (red), OHC-DC-junction$_1$ (green), and BM_{APJ} (blue), all relative to the sound pressure (in units of mm/Pa). The inset drawing shows the measurement locations for RL_3 (red circle), OHC-DC-junction$_1$ (green pentagon), and BM_{APJ} (blue diamond). **d–f** The phase responses corresponding to **a–c**. Note that in this animal the RL_3 results show broad tuning (panel **a**).

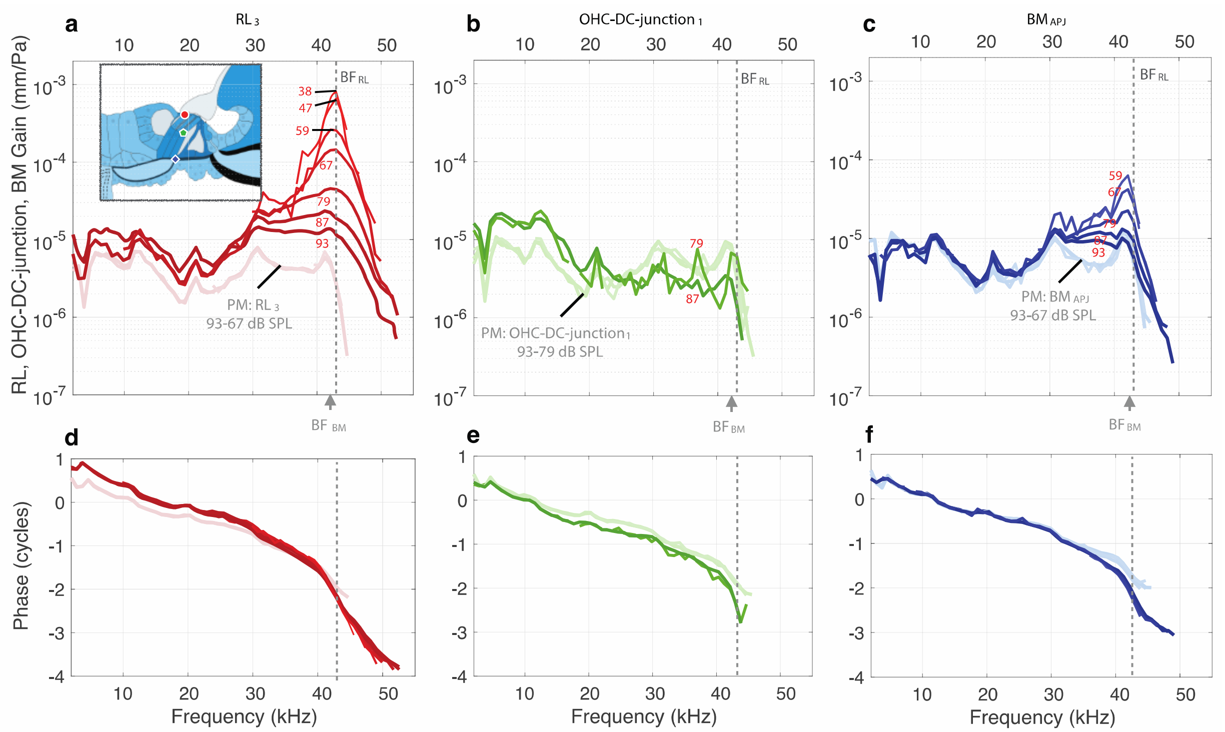

Supplementary Figure 8. Gerbil G645: **a–c:** In vivo (darker colors) and PM (faded colors) gains for RL_3 (red), OHC-DC-junction$_1$ (green), and BM_{APJ} (blue), all relative to the sound pressure (units of mm/Pa). The inset drawing in panel **a** shows the measurement locations for RL_3 (red circle), OHC-DC-junction$_1$ (green pentagon), and BM_{APJ} (blue diamond). **d–f:** The phase responses corresponding to **a–c**. Note that the available stimulus levels vary across the structures due to different signal-to-noise ratios.

Supplementary Note 4. Supplementary Figure 9 presents the motions at three radial points along the BM (BM_{PZ}, BM_{APJ}, and BM_{AZ}), normalized by the high-level BM_{APJ} gain (in dB), in animal G637.

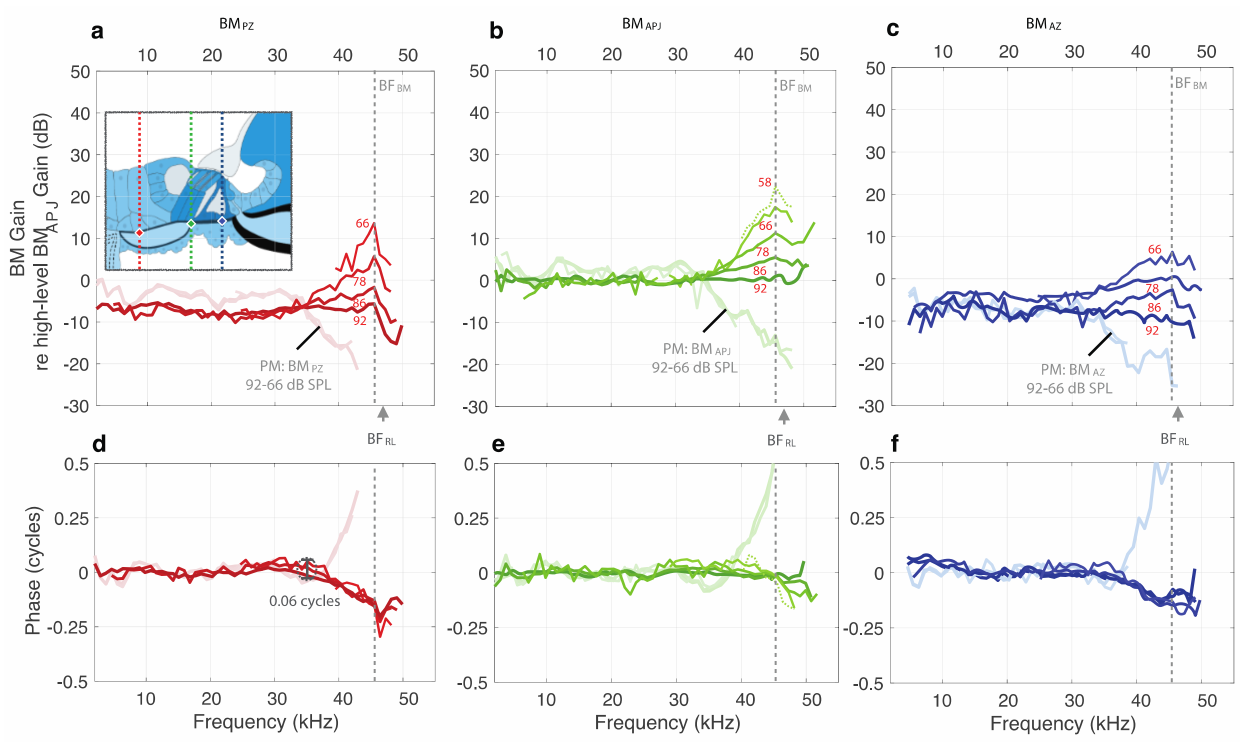

Supplementary Figure 9. Gerbil G637: **a–c:** In vivo and PM gains for BM_{PZ}, BM_{APJ}, and BM_{AZ}, respectively, normalized by the high-level BM_{APJ} gain. **d–f:** The phases corresponding to the magnitudes in **a–c**. The green dotted line shows the lowest-level BM_{APJ} data. The baseline high-level BM_{APJ} gain used for normalization the average of ten measurements made at 92 dB SPL.

Supplementary Note 5. On gerbil G637 we measured motion of the pillar-cell head (PC_H). In Supplementary Figure 10 we compare the in vivo BM_{APJ}, RL_1, and PC_H motions, normalized by the input sound pressure (left) and by the high-level BM_{APJ} gain (right.

Near BF, BM_{APJ}, RL_1, and PC_H had non-linear compression (a, b). RL_1 was more broadly tunned and had slightly higher gain than BM_{APJ} and PC_H. At frequencies more than 1/2-oct below BF, the motion of PC_H (magenta) was typically less than or equal to the motions of RL_1 (blue) and BM_{APJ} (green). PC_H had a broadly tunned dip of up to 10 dB near 30 kHz, while RL_1 has a sharp dip near 25 kHz. For RL_1, and PC_H, the gain continued to grow above BF and was higher than at BF.

From low frequencies to BF in all three measurements, there was a phase change of about 3 cycles (c), consistent with a traveling wave. PC_H phase was 0.15 cycles less than BM_{APJ} and RL_1 at 20 kHz (d). RL_1 phase led BM_{APJ} phase by 0.12 cycles near 25 kHz. The phase changed rapidly near BF for RL_1 and PC_H, but not as much for BM_{APJ}. This indicates a greater group delay for RL_1 and PC_H, than for BM_{APJ}, in this frequency region.

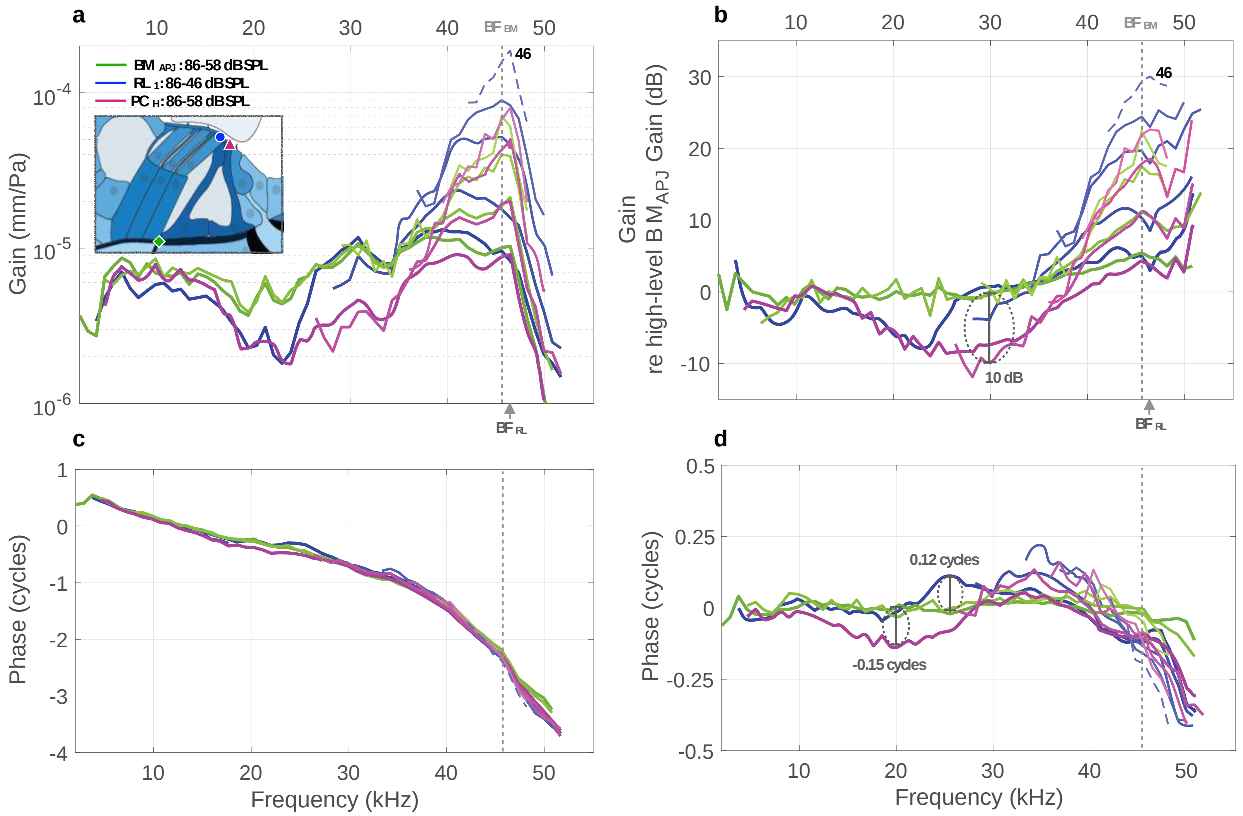

Supplementary Figure 10. a, b: In vivo gains of BM_{APJ}, RL_1, and PC_H normalized by sound pressure (left) and by averaged high-level BM_{APJ} gain (right) in Gerbil G637. The inset drawing shows the measurement locations for BM_{APJ} (green diamond) and RL_1 (blue circle), and PC_H (magenta tringle). **c, d:** The phases corresponding to the magnitudes in **a, b**. The blue dotted line shows the lowest-level RL_1 measurement (46 dB SPL); this level was not measured on BM_{APJ} and PC_H.
